# Effectiveness of sexual health interventions in reducing risky sexual behaviors among people living with HIV: a network meta-analysis and systematic review

**DOI:** 10.3389/fpubh.2026.1857629

**Published:** 2026-08-17

**Authors:** Yixuan Li, Yanxiao Gao, Nancy Reynolds, Qiaoyue Lu, Ziqi Qin, Yuqiong Duan, Pingwu Wang, Tao Liu, Ling Jie Cheng, Wenru Wang, Honghong Wang, Xueling Xiao

**Affiliations:** 1 School of Nursing, Hunan University of Chinese Medicine, Changsha, Hunan, China; 2 Xiangya School of Nursing, Central South University, Changsha, Hunan, China; 3 Shenzhen Institute of Advanced Technology, Chinese Academy of Sciences, Shenzhen, China; 4 Johns Hopkins School of Nursing, Baltimore, MD, United States; 5 National Perinatal Epidemiology Unit, Nuffield Department of Women’s and Reproductive Health, University of Oxford, Oxford, United Kingdom; 6 Alice Lee Centre for Nursing Studies, Yong Loo Lin School of Medicine, National University of Singapore, Singapore, Singapore; 7 Xiangya Center for Evidence-Based Nursing Practice and Healthcare Innovation: A Joanna Briggs Institute Affiliated Group, Changsha, Hunan, China

**Keywords:** condomless sex, multiple sexual partners, network meta-analysis, people living with HIV, sexual behavior

## Abstract

**Introduction:**

Numerous sexual health interventions have been employed to promote safer sexual practices among people living with HIV (PLWH). However, their effectiveness remains uncertain.

**Objectives:**

This review aimed to assess and rank the effectiveness of different sexual health interventions targeting risky sexual behaviors in PLWH.

**Methods:**

We systematically searched PubMed, Elsevier, the Web of Science, CINAHL, and the Cochrane Library for studies published between January 2010 and June 2025. Randomized controlled trials (RCTs) were included if they recruited PLWH aged ≥18 years and reported original quantitative data on condomless sex and multiple sexual partners across different groups. The network meta-analysis (NMA) was conducted within a Bayesian framework. We used a random effects model to calculate odds ratios (ORs) or standardized mean differences (SMDs) with 95% confidence intervals (CIs). Study quality was assessed using the Cochrane Risk of Bias (RoB) 2.0 tool, and the strength of the body of evidence was evaluated using the Grading of Recommendations, Assessment, Development and Evaluation (GRADE) framework.

**Results:**

A total of 34 eligible trials involving 12,967 PLWH and covering four types of sexual health interventions were included. In total, six trials reported outcomes on multiple sexual partners, but no significant effects were observed. A total of 33 trials reporting condomless sex were included in the network meta-analysis. Goal-oriented interventions ranked highest, with surface under the cumulative ranking curve (SUCRA) values of 0.903 and 0.828. Compared to standard care, goal-oriented interventions (OR: 0.54, 95%CI: 0.39 to 0.76) and safe sex skills training (OR: 0.65, 95%CI: 0.43 to 0.99) significantly reduced the likelihood of engaging in condomless sex. However, only goal-oriented interventions showed statistically significant superiority over control conditions in most subgroup analyses, including those stratified by country income level, intervention format and delivery mode, recruitment setting, and participant type.

**Conclusion:**

This review highlights the effectiveness of goal-oriented interventions in substantially reducing condomless sexual behaviors among PLWH. Such interventions should be prioritized to address the persistently high prevalence of risky sexual behaviors in this population, with important implications for preventing potential HIV recombination and other sexually transmitted infection (STI) co-infections, thereby reducing the overall health burden in this vulnerable group.

**Systematic review registration:**

Identifier CRD42023414692.

## Introduction

Risky sexual behaviors among people living with HIV (PLWH) remain a significant public health concern, as this population continues to be susceptible to sexually transmitted infections (STIs) and other co-infections despite the preventive benefits of antiretroviral therapy (ART). The “Undetectable = Untransmittable” (U=U) principle indicates that PLWH who achieve and maintain viral suppression do not sexually transmit HIV (1, 2). However, viral suppression does not prevent the acquisition or transmission of other STIs, and incident syphilis, hepatitis B virus infection, and hepatitis C virus infection have been documented among PLWH after achieving U=U (3). HIV superinfection also continues to pose clinical challenges, with incidence rates reaching up to 7.7% annually (4), further increasing health burdens and complicating treatment in this population. While biomedical prevention strategies such as PrEP and PEP are effective for HIV-negative individuals, their population-level impact remains limited due to constrained accessibility and coverage, particularly in low-resource settings (5). In this context, advocating for consistent safe sex practices, including consistent condom use and stable sexual partnerships, remains the most cost-effective and feasible approach. However, recent data indicate that the prevalence of risky sexual behaviors among PLWH remains alarmingly high, with rates ranging from 46 to 67% (6–8), highlighting the urgent need to identify effective interventions for this population.

Numerous interventions have been developed to address the high prevalence of risky sexual behaviors among PLWH, yet this issue persists. Some studies have implemented traditional health education interventions such as knowledge-based education, focusing on topics such as sexual negotiation and condom use (9). Others emphasize mental health among vulnerable populations, such as men who have sex with men (MSM) living with HIV and newly infected PLWH, by supporting stress coping and promoting safer sex through the alleviation of depressive and anxiety symptoms (10, 11). Other interventions adopt more complex approaches, often combining behavioral promotion strategies with improvements in interpersonal relationships (12, 13). However, these interventions have shown inconsistent effects, and some theoretically feasible methods are even discouraging (14).

Moreover, considerable inconsistency and heterogeneity exist among five published reviews summarizing the effectiveness of sexual health interventions for reducing risky sexual behaviors among PLWH. One meta-analysis confirmed a significant effect of “positive prevention” interventions in reducing the likelihood of condomless sex (15), while a 2021 meta-analysis suggested that psychological interventions may increase condom use in this population (16). However, another review found that such interventions did not reduce the number of sexual partners (17). Furthermore, two additional reviews lacked meta-analyses: One U.S. review supported evidence-based interventions (18), while the other, summarizing behavioral interventions for older adults living with HIV, reported conflicting results across six studies (19). Overall, the effectiveness of existing interventions remains uncertain, particularly as four of the five reviews were published over a decade ago and may now be outdated. Advances in intervention content, technology, and policy, such as eHealth interventions (20) and the “Undetectable = Untransmittable” campaign (21), may have further realigned intervention strategies.

In response, we conducted a network meta-analysis (NMA) to assess and compare the effectiveness of different sexual health interventions in reducing risky sexual behaviors among PLWH.

## Methods

Our systematic review and network meta-analysis were conducted in accordance with Cochrane methodology for systematic reviews of effectiveness and followed the PRISMA guidelines (22). The protocol was registered in PROSPERO under registration number CRD42023414692.

### Search strategies and selection criteria

We searched MEDLINE (PubMed), CINAHL (EBSCO), Excerpta Medica Database (Elsevier), the Cochrane Central Register of Controlled Trials (Ovid), and the Web of Science (Clarivate) from inception to 25 June 2025. We also searched the grey literature database (https://opengrey.eu/) up to 29 June 2025, and two international trial registers were searched—ClinicalTrials.gov and the World Health Organization’s International Clinical Trials Registry Platform—up to 20 June 2025. No restrictions were imposed on publication status or format. Grey literature records were screened using the same eligibility criteria as peer-reviewed publications and were not excluded solely because they had not been published as full-text journal articles. The search strategy was adapted for each included information source and is presented in Supplementary Appendix 1.

We included randomized controlled trials (RCTs) that recruited people living with HIV aged 18 years or older, evaluated sexual health promotion interventions, and reported condomless sex or multiple sexual partners. Studies involving both PLWH and individuals without HIV were also considered for inclusion if they reported original quantitative data specifically for the PLWH subgroup. For studies enrolling both participants aged ≥18 years and those aged <18 years, we contacted the corresponding authors to request outcome data specifically for the adult subgroup. Such studies were included only when data for participants aged ≥18 years were reported separately or were provided by the authors; otherwise, they were excluded. We excluded studies published before 2010 as the introduction of “treatment as prevention” and PrEP have transformed strategies for reducing risky sexual behaviors among PLWH. If a single study contained multiple independent data samples, we treated them as separate studies.

### Study selection and data extraction

All identified records were imported into EndNote 20 (Clarivate Analytics, Philadelphia, PA, USA), and duplicate records were removed. To better manage records and data throughout the review, study screening and data extraction were conducted using the Covidence platform (https://www.covidence.org/). Following a pilot test, both title and abstract screening and full-text eligibility assessment were conducted independently by two reviewers (QL and QQ) using Covidence. Disagreements were initially resolved through discussion between the two reviewers. If consensus could not be reached, a third reviewer (YL) adjudicated, with consultation with the wider review team when necessary. At the title and abstract screening stage, records were excluded only when they clearly failed to meet the eligibility criteria. When the information provided in the title or abstract was insufficient or eligibility remained uncertain, the full text was retrieved for further assessment. Any disagreements were resolved through discussion with YL, YD, and XX or through consultation with other co-authors as needed. Data from the included studies were independently extracted by three reviewers (PW, TL, and QQ) using a predefined standardized data extraction form. The extracted data were subsequently cross-checked, and discrepancies were resolved through discussion or consultation with YL or the wider review team. As mentioned above, data were collected from the included studies using a predefined data extraction form, which documented details including publication year, first author, country, sample size, mean age, proportion of female participants, study setting, intervention format, delivery personnel, and duration. In addition, the “Ycasd” software, a validated, reliable, and user-friendly tool for accurately extracting numerical data from graphical representations (23), was used to capture and scale numerical data reported only in figures and unavailable in the text or tables. The detailed results of data extraction from figures are shown in Supplementary Appendix 5, section 1.2. We contacted the authors by email when required data were not available. Studies were excluded if authors did not respond after three contact attempts within 7 days. The details of author contact records are presented in Supplementary Appendix 4. In addition, one cluster RCT was included in this network meta-analysis and analyzed together with individual RCTs, following the best practice recommended in the Cochrane Handbook (see Supplementary Appendix 5).

### Assessment of risk of bias and certainty of evidence

Eligible studies were critically appraised by two independent reviewers (QL and QQ) using the revised Cochrane Risk of Bias (RoB) 2.0 tool to assess the risk of bias (24). This tool evaluates the risk of bias in RCTs across five domains, including randomization, deviations from interventions, missing data, outcome measurement, and reporting. Overall risk of bias is rated as low if all domains are judged to be at low risk, as having some concerns if any domain raises concerns, and as high if one or more domains are judged to be at high risk or if multiple concerns compromise study validity. Disagreements were resolved by a third reviewer (YL) or group discussion. All studies, regardless of their methodological quality, underwent data extraction and synthesis. The strength of the body of evidence was assessed using the Grading of Recommendations, Assessment, Development and Evaluation (GRADE) framework, and the Confidence In Network Meta-Analysis (CINeMA) was used to evaluate the certainty of evidence (25).

### Data synthesis and analysis

The primary outcomes of this review were condomless sex and multiple sexual partners among PLWH. These outcomes included the proportion of participants engaging in condomless sex or having two or more sexual partners and the mean number of condomless sex acts or sexual partners within a specified time period. Further details on outcome data extraction and analyses are provided in Supplementary Appendix 5.

Sexual health interventions were classified according to the description of elements, emphasis, primary goals, and mechanisms provided in each study. In addition, previous research suggests that different control conditions can lead to different estimations of the effect size (26). Therefore, rather than aggregating all control groups into a single category, we classified them based on the specific descriptions outlined in each individual study. The characteristics and definitions of interventions are presented in Supplementary Appendix 6.

We performed a standard pairwise meta-analysis using the “metan” package in Stata version 15.1. We applied the DerSimonian and Laird random effects model for pairwise meta-analyses. Standardized mean differences (SMDs) were used for continuous outcomes, such as the mean number of condomless sex acts, while odds ratios (ORs) were used for binary outcomes, such as the proportion of participants engaging in condomless sex. The results were reported with 95% confidence intervals (CIs). Statistical heterogeneity was assessed using the I^2^ statistic, where an I^2^ > 50% indicated the presence of heterogeneity. In such cases, a random effects model was used to calculate the pooled effect sizes. Otherwise, a fixed effects model was applied. In addition, we assessed publication bias using Egger’s test, and a *p*-value of > 0.05 indicated no evidence of publication bias.

We performed a network meta-analysis (NMA) within a Bayesian framework. The “gemtc” package in R software version 4.2.3 was used to perform Markov chain Monte Carlo simulations. In the NMA, we evaluated two models: A fixed effects model and a random effects model. We configured the model with four chains, performing 100,000 iterations and 50,000 burn-in iterations. The convergence of the model was assessed through visual inspection of trace plots across the four chains and the Brooks–Gelman–Rubin diagnostic. We assessed the goodness of fit of each model to the data by calculating the posterior mean residual deviance. In addition, we used an arm-based data approach, estimating summary ORs for binary outcomes using a binomial likelihood and SMDs for continuous outcomes using a normal likelihood.

Before conducting the network meta-analysis, we evaluated the assumption of transitivity by considering clinical and methodological characteristics. As no standardized method exists to formally test transitivity across all pairwise comparisons, we examined key study characteristics that may influence effect estimates (27). Potential effect modifiers were identified through consultation with review team members with relevant clinical and methodological expertise. We visually compared mean age, the proportion of female participants, intervention duration, and geographic region across direct comparisons, while sample size was examined as a methodological characteristic. Other clinically relevant modifiers, including relationship status, ART adherence, suppression status, and depressive symptoms, could not be reliably assessed because of sparse and inconsistent reporting across comparisons.

In the network meta-analysis, local and global inconsistency was assessed using Stata (version 15.1) with the “mvmeta” and “network” packages (28). Local inconsistency was assessed using the node-splitting and loop-specific approaches, while global inconsistency was evaluated using the design-by-treatment interaction model. For heterogeneity assessment, we assumed a common heterogeneity variance for all treatment comparisons. We compared the posterior distribution of the estimated heterogeneity variance (τ^2^) with its predictive distribution, which was defined using empirical distributions of τ^2^ reported by Turner et al. (29) for binary outcomes and Rhodes et al. (30) for continuous outcomes. We classified heterogeneity as very low when it was below the 25th percentile, low when it fell between the 25th and 50th percentiles, moderate when it was between the 50th and 75th percentiles, and high when it exceeded the 75th percentile (31).

The mean rank and the surface under the cumulative ranking curve (SUCRA) were used to rank treatments for each outcome. Publication bias was assessed using comparison-adjusted funnel plots, and Egger’s test was performed to minimize the subjectivity of visual inspection. We conducted sensitivity analyses by excluding studies that focused on newly diagnosed HIV-positive individuals, studies with a sample size ≤20, studies including HIV serodiscordant couples, and studies with a high risk of bias. Network diagrams were generated using the ‘mvmeta’ package in Stata (version 15.1).

## Results

### Literature results

**Figure 1:**
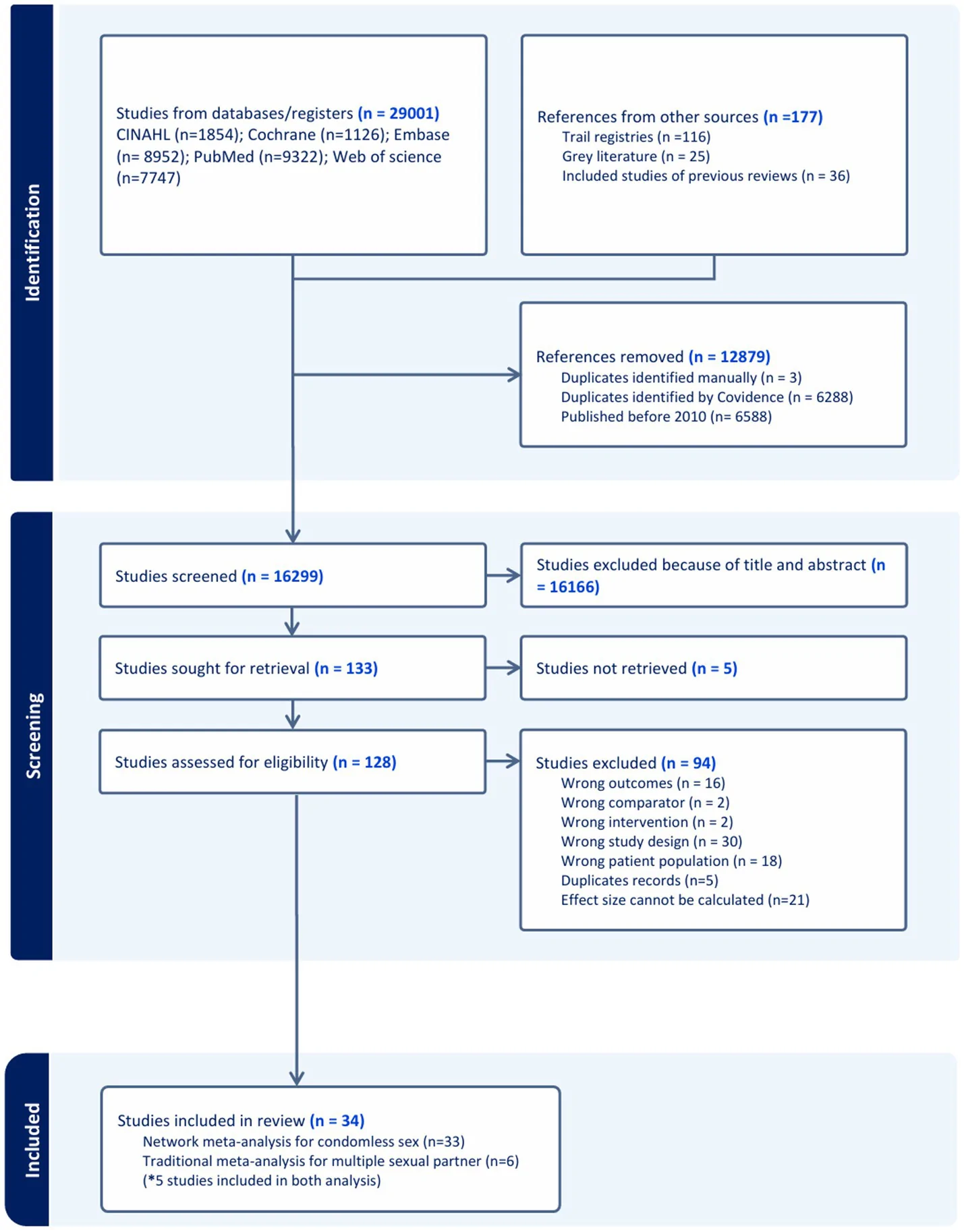
Study selection.

We identified 29,178 records, including 29,001 records from bibliographic databases and 177 records from other sources: Trial registries (*n* = 116), grey literature (*n* = 25), and previous relevant reviews (*n* = 36) (Figure 1). Of these, 6,291 duplicate records and 6,588 records published before 2010 were removed. We conducted full-text screening of 133 studies from the remaining16,299 records, of which 94 were excluded (Figure 1). A total of 34 studies, comprising 41 samples and including 12,967 PLWH, were eligible for inclusion in this review. In total, two studies reported both the proportion and frequency of condomless sex, 21 studies reported only the proportion of condomless sex, and 14 studies reported only the frequency of condomless sex. Of these, five studies also reported the number of sexual partners, while one additional study reported only the proportion of individuals with multiple sexual partners. Supplementary Appendices 2, 3 show the list of the included 34 RCTs and the studies that appeared to meet the inclusion criteria but were not included.

### Characteristics of the included studies

The included studies were published from 2010 to 2022. The average number of participants per study was 393 (ranging from 21 to 2,222), and the median intervention duration of the trials was 8 weeks (ranging from 1 to 48). The majority of studies (*n* = 21, 61.7%) recruited participants from North America, followed by Africa (*n* = 11,32.3%), China (*n* = 1, 2.9%), and Russia (*n* = 1, 2.9%). Approximately 30% of the studies focused on HIV-positive MSM, and five trials recruited only women living with HIV. In total, nine (26.4%) of the 34 studies employed online interventions, while the majority of the remaining studies focused on group-based interventions delivered in person by trained healthcare workers (see Supplementary Appendix 7).

### Traditional pairwise meta-analysis

The findings of the pairwise meta-analyses for condomless sex are presented in Supplementary Appendix 10. For proportion outcomes, goal-oriented interventions (GO) and education (Edu) showed statistically significant advantages in reducing the likelihood of engaging in condomless sex compared to standard of care (GO: OR: 0.50, 95%CI: 0.34 to 0.74; Edu: OR: 0.63, 95%CI: 0.50 to 0.80). However, other treatments did not show statistically significant differences compared to the control conditions. For frequency outcomes, we found a statistically significant difference in the two head-to-head comparisons: Goal-oriented intervention versus standard of care (SMD: -0.12, 95%CI: −0.20 to −0.03) and safe sex skills training versus waiting list (SMD: −1.05, 95%CrI: −1.55 to −0.55). I^2^ values ranged from 0 to 64.9% for the proportion outcome and from 0 to 93.8% for the frequency outcome, and we identified the direct comparisons with higher heterogeneity. Comparisons informed by a single trial were explicitly reported as having non-estimable heterogeneity. Egger’s tests yielded no statistically significant results for any of the comparisons, indicating no evidence of publication bias.

In addition, we conducted a meta-analysis of six studies reporting on multiple sexual partners (see Supplementary Appendix 18). Among these, three studies measured the proportion of individuals with multiple sexual partners, while the other three assessed the mean number of partners. Compared to control groups, the intervention groups showed no significant effects in reducing the likelihood of having two or more sexual partners (OR: 0.91, 95% CI: 0.70 to 1.17) or in decreasing the number of sexual partners (SMD: -0.03, 95% CI: −0.61 to 0.55).

### Network meta-analysis

**Figure 2:**
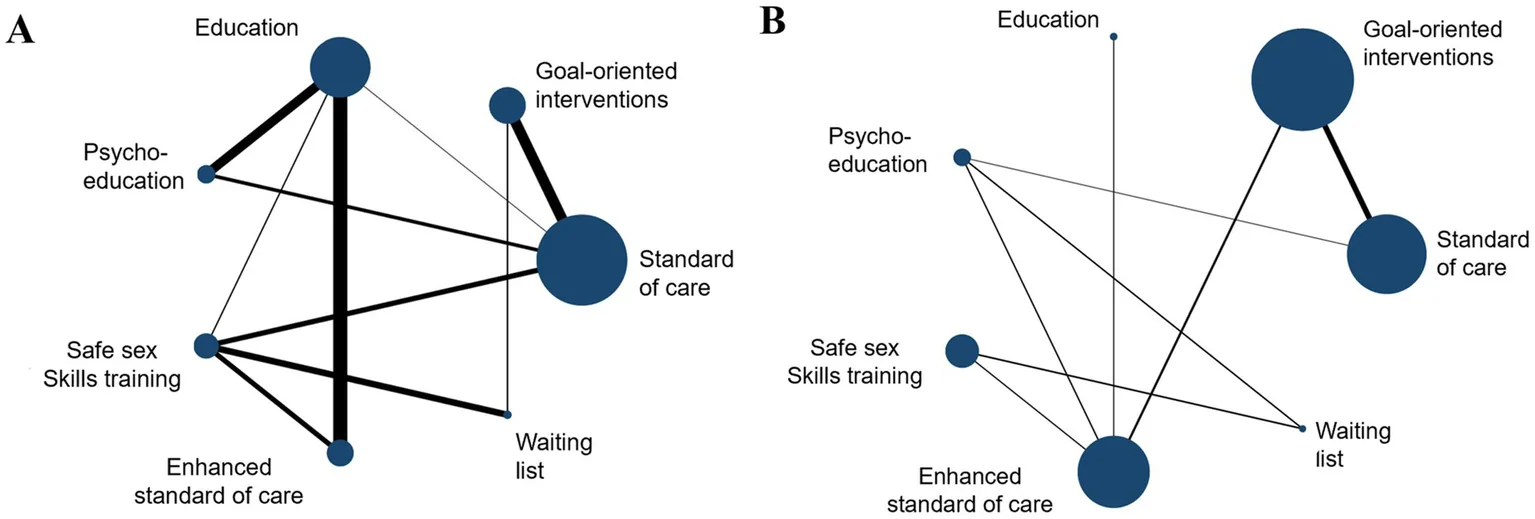
Network plot of eligible comparisons for the proportion of condomless sex **(A)** and the mean number of condomless sex acts in the past month **(B)**. Each node represents an intervention, with the size of the node proportional to the number of participants randomly assigned (i.e., sample size) and the thickness of the lines proportional to the number of trials for each pair of treatments.

**Figure 3:**
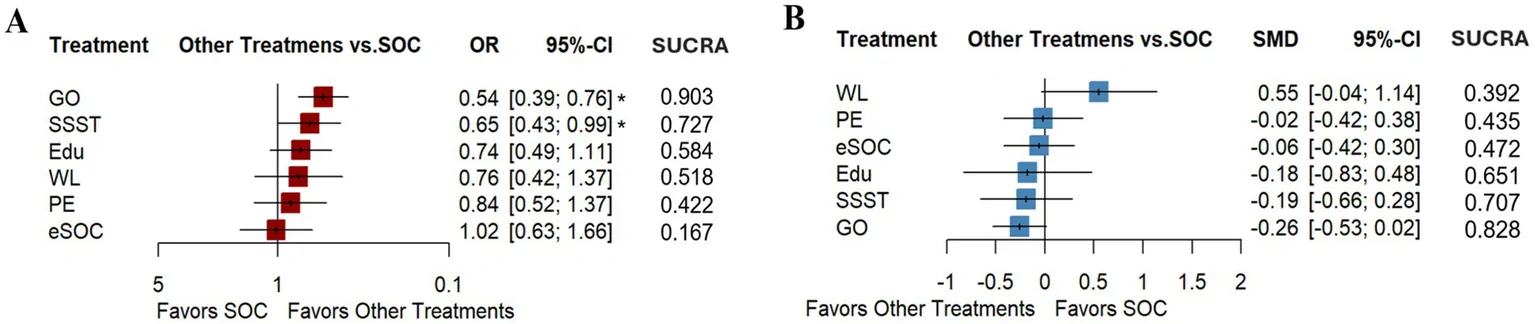
Forest plots of network meta-analysis for the proportion of condomless sex **(A)** and the mean number of condomless sex acts in the past month **(B)**. OR: Odds ratio; SMD: Standardized mean difference; SUCRA: The surface under the cumulative ranking curve; 95% CI: 95% confidence interval; *Significant results. GO: Goal-oriented intervention; WL: Waiting list; SOC: Standard of care; eSOC: Enhanced standard of care; PE: Psychoeducation; SSST: Safe sex skills training; Edu: Education.

**Figure 4:**
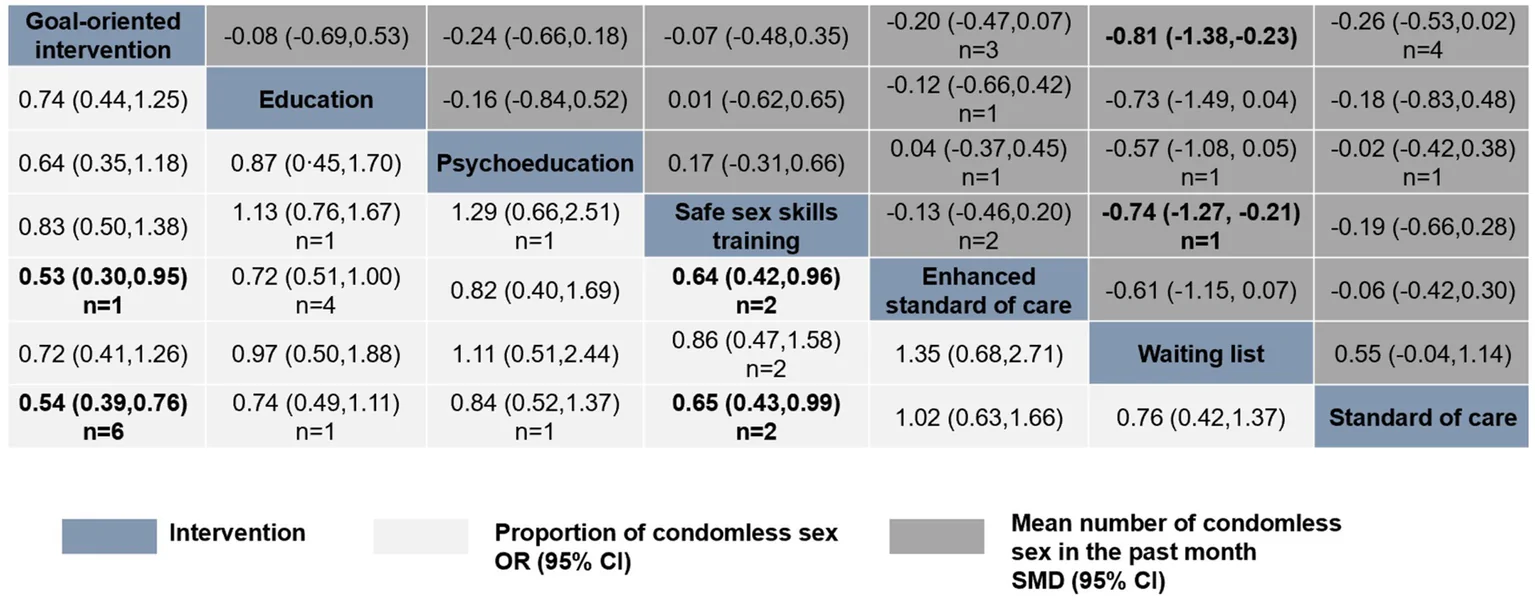
Results of mixed comparisons for the proportion and frequency outcomes of four sexual health interventions and three control conditions. Data are presented as ORs or SMDs (95% CI) for column-defined and row-defined treatments. The lower-left triangle presents the results for proportion outcomes (ORs), whereas the upper-right triangle presents the results for frequency outcomes (SMDs). ORs lower than 1 and SMDs lower than 0 favor the column-defining treatment. Significant results are presented in bold. *N* indicates the number of studies used for the comparison. OR, odds ratio; SMD, standardized mean difference; 95% CI, 95% confidence interval.

The network of eligible comparisons for condomless sex is shown in Figure 2. In total, four interventions and three control conditions formed two networks consisting of seven nodes each, resulting in 21 network comparisons. A total of 21 studies, including 8,288 participants, reported the proportion of PLWH engaging in condomless sex. Compared to standard of care, goal-oriented interventions showed a 46% reduction (OR: 0.54, 95%CI: 0.39 to 0.76) in the likelihood of engaging in condomless sex, and safe sex skills training showed a 35% reduction (OR: 0.65, 95%CI: 0.43 to 0.99), see Figures 3A, 4. For the proportion outcome, goal-oriented interventions had the highest SUCRA value (0.903), followed by safe sex skills training (0.727). For the frequency outcome, goal-oriented interventions also had the highest SUCRA value (0.828). However, none of the interventions showed a statistically significant difference compared to standard of care (Figures 3B, 4). Thus, the SUCRA values were interpreted as measures of relative ranking within the network rather than as independent evidence of intervention superiority. All network meta-analysis results reported were obtained using the random effects model, which provided a better fit compared to the fixed effects model (see Supplementary Appendix 9).

**Figure 5:**
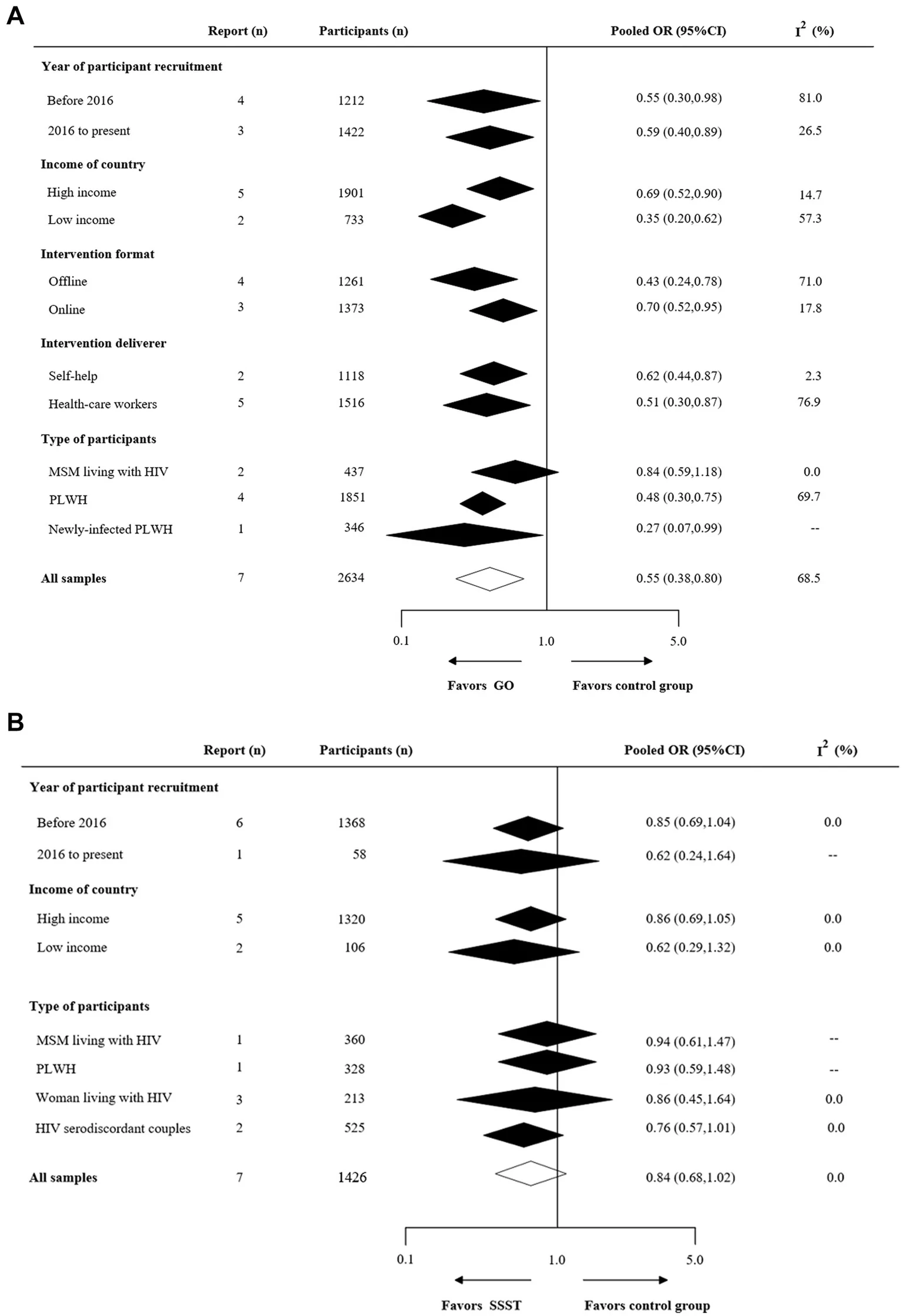
Subgroup meta-analysis of the effectiveness of goal-oriented interventions **(A)** and safe sex skills training **(B)** in reducing the prevalence of condomless sex. OR = odds ratio; PLWH = people living with HIV; MSM = men who have sex with men. GO = goal-oriented intervention; SSST = safe sex skills training.

We further conducted subgroup analyses for goal-oriented interventions and safe sex skills training. For goal-oriented interventions, subgroup analyses of seven studies found no statistically significant differences in the proportion outcome among PLWH across different country income levels, recruitment years, or intervention formats. However, the confidence intervals included the possibility of no difference among MSM living with HIV. The level of heterogeneity was substantially lower or absent in subgroups where participants were recruited after 2016 or from high-income countries, or where interventions were conducted online or delivered via self-help approaches (I^2^ range 0–26.5%; Figure 5A). For safe sex skills training, we found no heterogeneity among the seven studies that reported the proportion of condomless sex, but the estimates were no longer statistically significant in either the subgroup analyses or the overall sample (Figure 5B). Subgroup analyses for the frequency outcome are presented in Supplementary Appendix 12.

### Transitivity, heterogeneity, and inconsistency analysis

No definitive evidence was found to suggest violations of the transitivity assumption when evaluating potential effect modifiers across comparisons (see Supplementary Appendix 13). Very low heterogeneity was observed in the network for the proportion of condomless sex (tau = 0.23, IQR 0.36–0.42), whereas low heterogeneity was noted in the network for frequency outcomes (tau = 0.19, IQR 0.026–3.00), relative to the empirical predictive distributions (see Supplementary Appendix 14). Assessment of inconsistency is presented in Appendix 15. The design-by-treatment test indicated no significant global inconsistency in both networks (proportion: *p* = 0. 3,395; frequency: *p* = 0.3769). At the local level, both the proportion and frequency networks for condomless sex demonstrated good consistency, as neither the loop-specific nor the node-splitting approach identified significant inconsistency (all *p* ≥ 0.05).

### Publication bias

Supplementary Appendix 16 presents comparison-adjusted funnel plots for both proportion and frequency outcomes. The plots were approximately symmetrical, and Egger’s test yielded a *p*-value of 0.304 for proportion outcomes and a *p*-value of 0.224 for frequency outcomes. Thus, no evidence of publication bias was observed.

### Sensitivity analysis

In the sensitivity analysis, goal-oriented interventions consistently showed significant advantages over standard of care and maintained their ranking, except in analyses excluding studies involving newly diagnosed patients, those involving HIV serodiscordant couples, small sample studies, and studies rated as having a high risk of bias. However, for safe sex skills training, the confidence intervals included the possibility of no effect in the majority of sensitivity analyses (see Supplementary Appendix 17).

### Risk of bias and strength of evidence

In total, four studies were rated as having a low overall risk of bias, 11 studies had a high overall risk of bias, and the remaining studies were rated as having a moderate risk of bias (see Supplementary Appendix 8). CINeMA assessments indicated low to moderate confidence for most network comparisons. Therefore, confidence in the SUCRA-based treatment hierarchy was limited, and the rankings should be interpreted alongside the magnitude and precision of the relative effect estimates. GRADE ratings are integrated into Figure 4, with detailed assessments presented in Supplementary Appendix 19.

## Discussion

This network systematic review and meta-analysis assessed and ranked the effectiveness of different sexual health interventions for reducing risky sexual behaviors among PLWH. Our analysis indicates that goal-oriented interventions are likely the most effective approach, showing significant advantages over control groups in reducing both the likelihood and frequency of condomless sex. This effect remained consistent across various subgroups.

Overall, existing interventions emphasize setting measurable goals, enhancing knowledge, providing emotional support, and teaching practical skills to promote safer sexual practices. Despite the diverse focuses of existing interventions, a critical gap remains: None have explicitly integrated the U=U framework into their design or delivery (see Supplementary Appendix 6). Accurate and context-sensitive communication of the U=U principle is crucial. On the one hand, increasing numbers of national and international health agencies advocate for the widespread and accurate dissemination of the U=U message to ensure that PLWH are fully informed and can exercise their sexual health rights (32). On the other hand, studies have shown that avoiding the delivery of U=U-related information is associated with persistent misconceptions about its meaning and implications (33). Such misunderstandings may result in PLWH receiving inaccurate or incomplete information, which can lead to an underestimation of the risks associated with engaging in condomless sex. Therefore, integrating the U=U principle into intervention design and delivery could enhance both the effectiveness and contextual relevance of sexual health interventions.

We found that both goal-oriented interventions and safe sex skills training reduced the likelihood of condomless sex compared to standard of care. However, in subgroup analyses, the effect estimates for goal-oriented interventions remained statistically significant in most subgroups, whereas the benefits of safe sex skills training became less pronounced. This finding may indicate that the apparent effectiveness of safe sex skills training is largely influenced by indirect comparisons. Furthermore, there is no statistically significant difference among the four sexual health interventions in either the proportion or frequency outcomes. Possible explanations include the limited number of trials evaluating education, psychoeducation, and safe sex skills training, as well as the reliance on comparative results for intervention conditions, which were mostly derived from indirect comparisons.

The potential benefit of goal-oriented interventions is consistent with previous evidence supporting the use of goal-setting strategies to promote health behavioral change (34). Sexual behaviors among PLWH involve a complex decision-making process characterized by unpredictability and instability and influenced by multiple factors. Goal-oriented interventions offer unique advantages in addressing these challenges, primarily by incorporating two key components: Goal setting and action planning. Setting clear and specific goals tailored to the individual’s circumstances, such as aiming to reduce the number of sexual partners over the next six months or practicing refusal skills to avoid unprotected sex in high-risk situations every week, can effectively stimulate intrinsic motivation (35, 36) and improve adherence to action plans, thereby supporting behavioral change (37, 38). This benefit may be attributed to the goal-setting process, through which individuals gain a clearer understanding of the risks they face and increase their acceptance of specific behavioral changes needed. In addition, detailed action plans help reduce the challenges associated with behavioral change. These plans may include steps such as obtaining and using condoms correctly and negotiating safer practices with partners.

However, despite these benefits, goal-oriented interventions may face challenges in effectively managing condomless sex among MSM living with HIV. One potential reason for this limitation is the compounded self-stigma associated with both HIV status and MSM identity, which goal-oriented interventions may not fully address (39). In addition, the dynamics of sexual relationships and risk behaviors among MSM communities are often complex and changeable (40). Therefore, sexual health interventions among MSM living with HIV may require more personalized and real-time monitoring approaches.

According to our SUCRA results for both proportion and frequency outcomes, education and safe sex skills training ranked second and third, respectively, among the four sexual health interventions. Safe sex skills training showed significant advantages over control conditions in reducing both the proportion and frequency of condomless sex and was most likely the second most effective intervention. However, the statistical estimates were not stable in subgroup and sensitivity analyses. A similar pattern was observed for educational interventions, which remain widely used and are incorporated into sexual health service guidelines for people living with HIV (41). These approaches may lack consideration for the unique needs of different individuals. Moreover, previous reviews have highlighted that tailored interventions are more effective than non-tailored interventions (42), offering greater sensitivity in addressing the needs of vulnerable and socially marginalized populations (43). The instability of these findings may be attributable to the limited number of included studies. Therefore, more large-sample, high-quality RCTs are needed to identify the effectiveness of educational and skills training interventions.

Interestingly, psychoeducation had poor SUCRA values in both networks, even ranking below some control conditions. This finding contrasts with that of a previous review, which reported that psychological interventions were effective in reducing sexual risk behaviors among PLWH (16). This discrepancy may stem from differences in the definition of psychological interventions. Zhang et al. (16) adopted a broader definition, whereas the psychoeducation interventions included in our analysis specifically focused on emotional support and coping strategies. Furthermore, some studies have reported no association between depression and risky sexual behaviors (44), with evidence suggesting that PLWH experiencing severe depression may be less inclined to engage in risky sexual behaviors due to the preponderance of debilitating neurovegetative symptoms (45). Thus, psychoeducation may not be a universally effective intervention, and individualized assessments are likely necessary before its application.

Based on the available evidence, goal-oriented components, particularly goal setting and action planning, may be considered when designing sexual health interventions to reduce condomless sex among PLWH. However, this recommendation should be interpreted cautiously because the favorable ranking of goal-oriented interventions was supported by low- to moderate-certainty evidence, and their comparative superiority over other active interventions was not established. However, there are significant gaps in the evidence: No interventions explicitly address the U=U (“Undetectable = Untransmittable”) message, potentially leaving patients unaware of how to engage in safer sexual practices. In addition, there is a lack of studies targeting the reduction of multiple sexual partners, and it remains uncertain whether intervention effectiveness varies across different populations and follow-up durations or whether other potentially effective interventions exist. Therefore, further research with robust study design, extended follow-up periods, targeted evaluation of interventions aimed at reducing the number of sexual partners, and direct comparisons of alternative strategies is needed. Equally important is ensuring the precise communication of the U=U principle within healthcare delivery.

### Limitations

To the best of our knowledge, this is the first network systematic review and meta-analysis to investigate the effectiveness of different sexual health interventions for reducing risky sexual behaviors among PLWH, offering practical insights for HIV healthcare providers. However, our review has some limitations. First, nearly one-third of the included studies were rated as having a high risk of bias, indicating that our results should be interpreted with caution. This may also reflect the influence of uncontrolled factors, such as personal motivation, cultural background, and social support, as well as the inherent difficulty of implementing double-blinding in sexual health interventions. Second, the limited number of studies that reported multiple sexual partners prevented us from conducting a network meta-analysis. Third, differences in populations, such as newly diagnosed individuals and HIV serodiscordant couples, may affect the reliability of the evidence. Despite this, the subgroup analyses and sensitivity analyses did not provide substantial evidence suggesting violations of the transitivity and consistency assumptions across different populations. Fourth, the clinical significance of the statistically significant findings remains uncertain because no established threshold exists for a clinically meaningful change in condomless sex, and heterogeneity in baseline risk, outcome measurement, and follow-up limited the interpretation of the pooled estimates in absolute terms. Finally, we were unable to conduct subgroup analysis by follow-up time as only two studies followed participants for more than one year, making it difficult to assess the long-term sustainability of intervention effects.

## Conclusion

The results of this review have important implications for the prevention of further co-infection among PLWH, including HIV superinfection and other STIs. Goal-oriented interventions, particularly those incorporating personalized goal setting and planning, showed the greatest promise for reducing the likelihood of engaging in condomless sex among PLWH. However, their relative ranking should be interpreted cautiously given the uncertainty of the effect estimates and the generally low-to-moderate certainty of evidence. These findings support the consideration of goal-oriented components in sexual health services for PLWH, with appropriate adaptation to individual circumstances and accurate communication of U=U principles. Theoretically, our findings suggest that structured goal setting and self-regulation may be important mechanisms through which sexual health interventions promote behavioral change among PLWH, although these mechanisms require direct empirical testing. Future studies should use adequately powered, theory-driven RCTs with standardized outcome definitions, longer follow-up, systematic measurement of key effect modifiers, and confidential, participant-centered data-collection methods, supplemented by objective STI outcomes where feasible, to minimize social-desirability bias and establish the clinical relevance of intervention effects.

## Data Availability

The original contributions presented in the study are included in the article/Supplementary material, further inquiries can be directed to the corresponding author.
